# Improvement of Polymer/Metal Adhesion Using Anodizing Treatment and 3D Printing Process

**DOI:** 10.3390/polym17030299

**Published:** 2025-01-23

**Authors:** Seung Wan Ryu, Dong Hyun Kim, Wonhwa Lee, Jin-Yong Hong, Young-Pyo Jeon, Jea Uk Lee

**Affiliations:** 1 Hydrogen&C1 Gas Research Center, Korea Research Institute of Chemical Technology (KRICT), 141 Gajeong-ro, Yuseong-gu, Daejeon 34114, Republic of Korea; skyzoop@naver.com (S.W.R.); jyhong@krict.re.kr (J.-Y.H.); 2 Department of Advanced Materials Engineering for Information and Electronics, Integrated Education Institute for Frontier Science and Technology (BK21 Four), Kyung Hee University, 1732 Deogyeong-daero, Giheung-gu, Yongin-si 17104, Republic of Korea; spy04032@khu.ac.kr (D.H.K.); lwh980304@khu.ac.kr (W.L.); 3 Advanced Materials and Chemical Engineering, University of Science and Technology (UST), 217 Gajeong-ro, Yuseong-gu, Daejeon 34113, Republic of Korea

**Keywords:** polymer–metal joints, aluminum anodizing, porous surface, injection molding, 3D printing

## Abstract

Joining materials with different physicochemical properties presents significant challenges. This study investigates the one-step anodization of aluminum in a mixed phosphoric acid and hydrogen peroxide solution, followed by the direct injection molding of polymer resin to enhance joint properties. The anodizing treatment is performed at constant electrical current with phosphoric acid solutions of various concentrations. Phosphoric acid anodizing enables the formation of 3D channeling pore structure with micropits and uniform nanopores on the aluminum surface. Hydrogen peroxide acts as an oxidizing agent and promotes the dissolution reaction, thereby increasing the size of the nanopores. Larger pores facilitated the penetration of polymer resin into the aluminum oxide layer during injection molding, resulting in bonding strengths up to 40.34 MPa. This improvement is substantial when compared to the bonding strengths achieved through conventional injection molding processes. These results highlight that the increase in nanopore size due to hydrogen peroxide addition played a critical role in enhancing the bonding strength, as it facilitated better penetration and interlocking of the polymer resin within the anodized aluminum layer. Furthermore, a three-dimensional (3D) printing process was able to join polymer resins to the anodized aluminum surface, where the larger nanopores with the addition of the hydrogen peroxide is more beneficial to the bonding strengths than the direct injection molding is. This alternative approach addresses the environmental issues associated with the use of Cr(VI)-based anodizing solutions and the lightweight composites with applicability to various industries that could be produced using this method.

## 1. Introduction

In recent years, the use of metal–polymer joints to improve the properties of metals has gained popularity. However, joining materials with different physicochemical properties presents significant challenges [1]. Various methods are employed to join metals and polymers, including adhesive bonding, self-piercing riveting (SPR), and friction stir welding (FSW). In the case of adhesive bonding, the accurate selection of adhesives based on the properties of the metal and the polymer is essential. The bonding strength is largely dependent on the properties of the adhesives. For the mechanical SPR and FSW methods, there is a need for expensive equipment, which may affect the integrity of the materials owing to the weight of the additional parts and the physical forces that act directly on the metal [2,3,4,5].

Recently, direct joining methods and direct injection molding have been used to bind metals and polymers without introducing adhesives or additional parts. Welding is the most common direct joining method. Metals and polymers can be joined by applying localized heat to a laminated surface, melting the polymer. This heat can be delivered through laser radiation [6,7,8], ultrasonic radiation [9,10], or friction heating [11,12]. Taki et al. investigated the joining of a laser-ablated metal and polymer [13]. They applied direct joining methods after enhancing the roughness of the metal by surface treatment, and achieved a joint strength of 12 MPa. However, even higher bonding strengths are often required for industrial applications.

Alternatively, direct injection may be applied to join different materials without the use of additional substances; for example, a polymer can be injected into surface-treated aluminum metal under high temperature and pressure. The most widely used surface treatment is anodizing, in which an aluminum oxide layer with a controlled thickness is formed by electrochemical treatment in an acidic solution. Earlier reports of aluminum surface treatment predominantly involved the use of a Cr(VI)-sulfuric acid anodizing solution [14]. This method effectively forms an oxide layer and ensures high bonding strength and durability. However, the industrial use of Cr(VI)-based solutions has declined due to stringent global regulations prompted by environmental concerns.

Consequently, considerable research has focused on the use of alternative anodizing solutions to modify metal surfaces and enhance polymer–metal adhesion. Sulfuric acid, phosphoric acid, and oxalic acid have been proposed as potential alternatives [15,16,17,18]. In particular, aluminum oxide anodized with oxalic acid exhibits linear pores, and it has been successfully used to produce anodic aluminum oxide with a well-ordered pore structure [19,20,21]. Polymer–metal adhesion relies on the interlocking effect between the two materials. However, linear pores produced using oxalic acid do not facilitate this interlocking. Aluminum anodizing with sulfuric acid has been widely applied. The low pH enables a high oxide generation rate, facilitating minimal pore defect formation and excellent oxide layer thickness [22]. However, a large number of water molecules become trapped within the anodic oxide layer because of the influence of the ambient environment [23,24]. Consequently, when a polymer resin is injected into aluminum at a temperature above its melting point, these trapped water molecules are released inside the anodic film and they adversely affect the adhesive properties. Furthermore, when anodizing is performed exclusively with sulfuric acid, it is challenging to form a ‘bird’s nest’-like morphology. This morphology, comprising microsize pits with nanopores, facilitates the penetration of polymer resin into the aluminum surface pores [25,26]. The chemical properties and surface structure of the aluminum surface play an integral role in enhancing the bonding strength between the metal and the polymer. The aluminum surface, which contains a large number of hydroxyl groups, can chemically interact with the polymer resin to increase the bonding strength. The proportion of hydroxyl groups on the aluminum surface is larger when phosphoric acid is used as the electrolyte instead of sulfuric acid [27]. Moreover, because the pores act as an inlet to facilitate the penetration of the polymer resin into the aluminum oxide layer, the increased pore size can enhance penetration. Many studies have achieved an increased pore size by dipping aluminum samples in a phosphoric acid solution after anodization [28,29]. However, this additional experimental steps are not favorable for industrial implementation.

**Figure 1:**
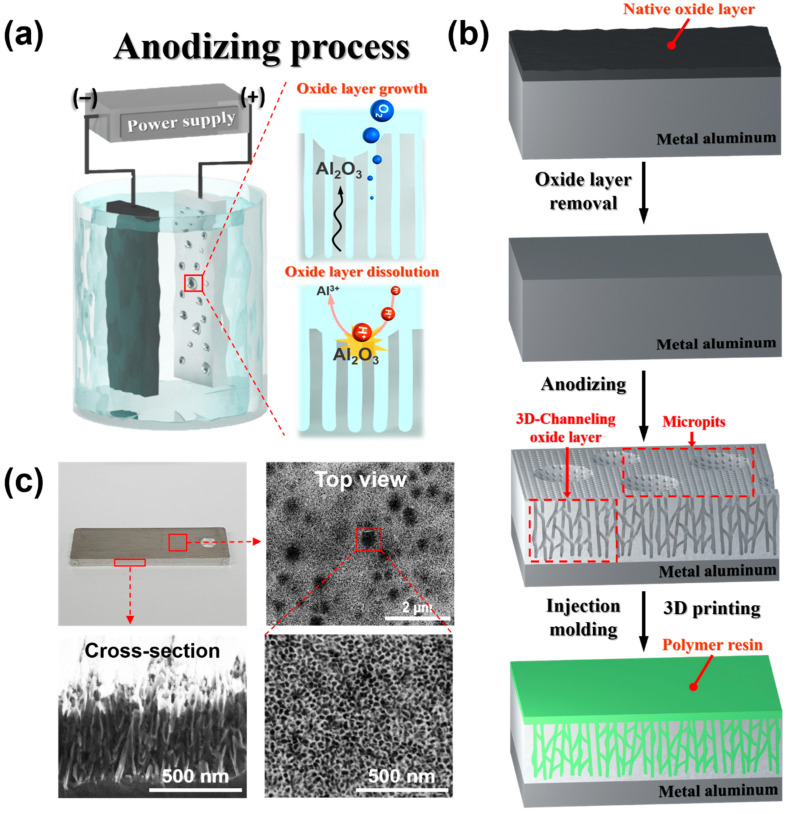
Illustration of the formation of nanopores on the surface of aluminum. (**a**) Anodizing process, (**b**) formation of nanopores, 3D-channeling oxide layer obtained as a result of anodization, and direct joining of polymer resin on aluminum surface via injection molding or 3D printing, and (**c**) top and cross-section SEM images of the anodized sample.

This study investigates the single-step anodizing treatment of aluminum in a mixed phosphoric acid and hydrogen peroxide solution. This is followed by direct injection molding and three-dimensional (3D) printing of polymer resin to achieve joint properties suitable for industrial use (Figure 1). First, the anodizing treatment is performed at a constant electrical current using various concentrations of phosphoric acid to determine the optimal concentration. Phosphoric acid anodizing leads to the formation of 3D channeling pore structure with uniform nanopores on the aluminum surface, and micropits were observed. Hydrogen peroxide acts as an oxidizing agent and various concentrations are added to the phosphoric acid solution. The generation of additional hydrogen ions from hydrogen peroxide during the electrochemical reaction further activates the dissolution reaction, thereby increasing the size of the nanopores. The larger pore size facilitates the penetration of the resin into the aluminum oxide layer, which results in the outstanding bonding strengths up to 40.34 MPa, one of the highest values reported for polymer/metal joints. Further, by innovatively utilizing 3D printing to join polymer resins with anodized aluminum surfaces, this research eliminates the need for heavy molds and equipment, showcasing a groundbreaking approach to achieving superior bonding through optimized nanopore sizes. The 3D-printed polymer/metal joint sample with larger nanopores, achieved by adding hydrogen peroxide, exhibits a higher bonding strength of 8.50 MPa. This is greater than that of joint samples with a smaller nanopore distribution.

## 2. Experimental

### 2.1. Materials

Aluminum alloy (AA6062) with a chemical composition of 97.6% Al, 0.4% Si, 0.8% Mg, 0.4% Cu, 0.7% Fe, and 0.1% Mn was purchased from Blue Science Corporation (Daejeon, Korea). Phosphoric acid (85 wt%), sulfuric acid (95%), and stearic acid (95%) were purchased from Sigma-Aldrich. Sodium hydroxide (98%) was purchased from Alfa Aesar (Ward Hill, MA, USA). Metal degreasing agent C-4000 was purchased from Kyunggichem Corporation (Gyeonggi-do, Korea). A graphite sheet (150.0 × 50.0 × 3 mm) was supplied by Daeyoung Carbon (Seoul, Korea) and polybutylene terephthalate (PBT, 1500G15) was purchased from Samyang Corporation (Sejong-si, Korea). Polylactic acid filament (PLA, diameter ~1.75 mm) was purchased from Cubicon Corporation (Daejeon, Korea).

### 2.2. Pretreatment of Aluminum Alloy Sheets for Anodizing

Aluminum alloy sheets (40.0 × 12.0 × 3 mm) were immersed in ethanol and acetone. The sheets were sonically cleaned sequentially in acetone, ethanol, and deionized water for 10 min. The cleaned aluminum sheets were pretreated in acidic and alkali solutions of C-4000, NaOH, and H_2_SO_4_. In particular, the organic residue on the aluminum surface was removed for 2 min at 50 °C using C-4000 solution (5 wt%). The native oxide layer was removed at 40 °C for 2 min using NaOH solution (5 wt%). Finally, the remaining organic residue and oxide layer were removed at 60 °C for 3 min in H_2_SO_4_ (10 wt%). The sheets were washed with deionized water between each pretreatment step to remove the residual solution from the prior step.

### 2.3. Anodizing Treatment of Aluminum Alloy Sheets

The pre-treated aluminum sheets were anodized in the various anodizing solutions under constant electrical current conditions, thereby facilitating the formation of a nanoporous aluminum oxide layer. Phosphoric acid anodizing solutions with various concentrations (5, 7, and 15 wt%) were prepared, and the aluminum samples treated with these solutions were denoted as P5, P7, and P15, respectively. Hydrogen peroxide was added to the optimal phosphoric acid anodizing solution (7 wt%) at various concentrations (100, 250, and 500 mM), and the aluminum samples treated with these solutions were denoted as P7/H100, P7/H250, and P7/H500, respectively. During anodization, the aluminum and graphite sheets acted as the anode and cathode, respectively, and the anodizing solution served as the electrolyte solution. The anode and cathode were separated by 10 mm within the electrochemical cell, while the electrolyte was maintained at 30 °C and stirred at 100 rpm with a magnetic bar. The aluminum sheets were anodized at a constant current density of 1.5 A/dm^2^ for up to 15 min using a DC power supply (IT6952A, KMI system, Seongnam, Korea). The samples were rinsed with deionized water and dried in an oven at 80 °C for 15 min.

### 2.4. Insert Injection Molding of Polymer Resin on Anodized Aluminum

The polymer resin and anodized aluminum samples were joined via a typical injection molding process. The anodized aluminum sheet (40.0 × 12.0 × 3.0 mm^3^) was inserted into the mold, and the PBT polymer (40.0 × 12.0 × 3.0 mm^3^) was injected using a commercial injection molding machine (ROBOSHOT α-S100iA, FANUC, Yamanashi, Japan). The molding machine used had a screw diameter of 22 mm. Given that joining strength results can vary with changes in molding conditions, we initially determined the optimal molding conditions that yield the highest joining strength through a review of the literature [30] and a series of experiments (Figure S1). The insert injection molding conditions employed in this study are outlined as follows: A nozzle temperature of 250 °C and an injection speed of 10 mm/s. The holding pressure was set at 40 MPa. The mold temperature was maintained at 140 °C throughout the molding process. To precisely measure the differences in bonding strength of polymer/metal joint samples prepared under various anodizing treatment conditions, an end-to-end butt joint with no groove was used.

### 2.5. 3D Printing of Polymer Resin on Anodized Aluminum

The polymer resin and anodized aluminum samples were also joined via a 3D printing process. A 3D fused deposition modeling (FDM) printer (3D Factory, Ulsan, Korea) was used for printing the PLA polymer filament onto the anodized aluminum samples. After securing the anodized aluminum sheet (40.0 × 12.0 × 3.0 mm^3^) to the 3D printing bed, polymer filaments were 3D-printed onto it in a T-butt joint geometry (40.0 × 12.0 × 3.0 mm^3^). Given that joining strength results can vary with changes in 3D printing conditions, we initially determined the optimal printing conditions that yield the highest joining strength through a series of experiments (Figure S2). The 3D printing conditions employed in this study are outlined as follows: The nozzle diameter of the FDM unit was 1.0 mm, and nozzle and bed temperature was set at 230 and 150 °C, respectively, during FDM process. Printing speed was at 300 mm/min and layer thickness was 0.5 mm. The infill density was set at 100%.

### 2.6. 3D Characterization

The morphology of the anodized aluminum sheets was observed using field-emission SEM (FE-SEM) (Tescan, Mira 3 LMU FEG, Brno, Czech Republic). The pore size and its distribution were calculated using the open-source ImageJ software (version 1.54m 5), based on FE-SEM images and following conventional pore size characterization methods [31]. An Alpha-Step profiler was used to measure the roughness of the aluminum sheets according to ISO 4287 (1997) standard [32], where Alpha-Step images were obtained using a Bruker DektakXT Stylus Profiler (10th generation system, Karlsruhe, Germany). More than three specimens for each sample and conducted SEM observations and roughness measurements repeatedly. The X-ray diffraction (XRD) analysis was conducted using a Rigaku D/Max 2200V X-ray diffractometer (Tokyo, Japan). The surface chemistry was measured using X-ray photoelectron spectroscopy (XPS, AXIS NOVA, KRATOS, Manchester, UK), which was conducted in the wide and small scan modes using a monochromatic Al-Kα (15 KeV) X-ray source. The adhesive properties of the polybutylene terephthalate (PBT)/aluminum and PLA/aluminum joint samples were measured in samples with a bond area of 1.2 × 0.3 mm using a tensile strength measurement instrument (Instron 5569, Norwood, MA, USA) in the progressive mode according to procedures described by ASTM: KS M 3705-2015 [33]. The average bonding strength value was determined from eight different samples for each polymer/metal joint.

## 3. Results and Discussion

### 3.1. Surface Morphology of Anodized Aluminum Oxide Layer

The anodizing process was conducted using different ratios of phosphoric acid to hydrogen peroxide, as described herein. The characteristics of the aluminum surface and the pore sizes resulting from each treatment combination were analyzed carefully using scanning electron microscopy (SEM). Phosphoric acid facilitated the reaction between aluminum and oxygen ions at the metal/oxide interface, thereby forming an aluminum oxide (Al_2_O_3_) layer (Figure 1a). The oxygen gas produced from a side reaction was released through the barrier layer. The use of phosphoric acid resulted in the formation of unique serrated structures in the inner wall of the pores during oxygen release (Figure 1b,c) [34]. The joining of metal and polymer materials was facilitated by this 3D channeling pore structure of the oxide layer, which induced the interlocking of the polymer from within. This led to superior joint formation compared to that of a linear oxide layer.

**Figure 2:**
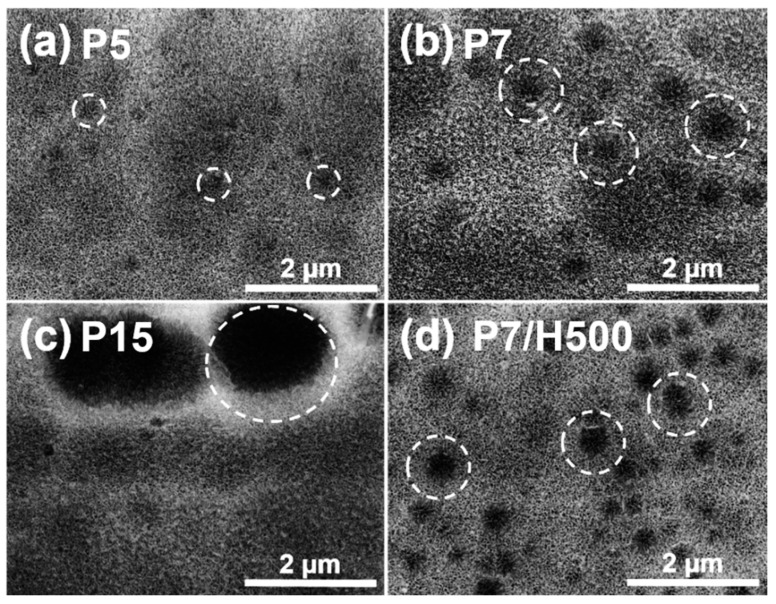
SEM images of anodized aluminum surface with micropits (white dotted circles). (**a**) P5, (**b**) P7, (**c**) P15, and (**d**) P7/H500.

Figure 2 shows SEM images of aluminum samples anodized in the mixed phosphoric acid and hydrogen peroxide solution. The SEM image of sample P5 (aluminum sample with 5 wt% phosphoric acid anodizing treatment) shows that micropits (diameter = 0.2 μm) and unevenly sized nanopores were formed (Figure 2a). The submicron-sized pits on the aluminum surfaces were generated following sequential pretreatment with NaOH and H_2_SO_4_ (Figure S3). The SEM image of sample P7 (aluminum sample with 7 wt% phosphoric acid anodizing treatment) revealed larger micropits (~1 μm) and nanopores with diameters in the range of ~20–30 nm (Figure 2b). Considerably larger micropits (~3 μm) were formed in sample P15 (aluminum sample with 15 wt% phosphoric acid anodizing treatment) (Figure 2c). The hydrogen and phosphate ions generated from phosphoric acid contributed to the formation of nanopores and etched the aluminum surface, thereby enlarging the micropits during the anodizing treatment. Wang et al. compared the use of phosphoric acid, ammonium phosphate, and nitric acid solutions, and they confirmed that both hydrogen and phosphate ions are critical in the etching process [35]. Further, they reported that the etching rate increased with increasing phosphoric acid concentration. In our study, sample P15 was treated with excess phosphate, which led to an increased etching rate and extensive micropits formation. In general, increasing the anodizing time or applying more intense anodizing conditions, such as the use of higher concentrations of phosphoric acid, can lead to extended etching of aluminum. This can cause the collapse of the top part of the oxide layer during anodizing, thereby increasing the size of micropits; this extreme modification may act as a defect instead of facilitating the adhesion of the polymer resin. Therefore, the conditions used to treat sample P7 were considered to be the most appropriate in the size and uniformity of the pores to enhance polymer/metal adhesion. Therefore, this condition was used in the following steps.

**Figure 3:**
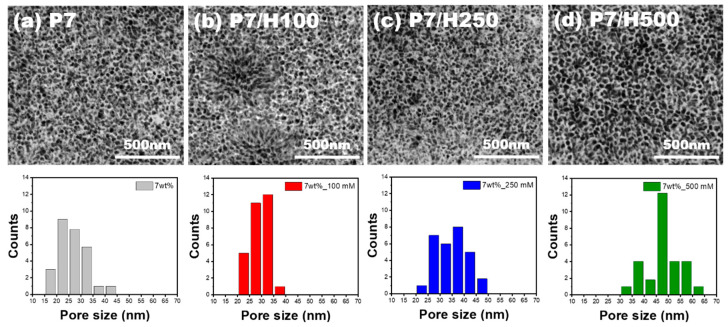
Magnified SEM images of anodized aluminum surfaces and size distribution of nanopores. (**a**) P7, (**b**) P7/H100, (**c**) P7/H250, and (**d**) P7/H500.

SEM images of samples P7/H100, P7/H250, and P7/500 (aluminum samples with 7 wt% phosphoric acid anodizing treatment with 100, 250, and 500 mM hydrogen peroxide) are shown in Figure 2d and Figure S4. Although the size of the micropits was dependent on the phosphoric acid concentration, it was not affected by adjusting the amount of hydrogen peroxide. Thus, the sizes of the micropits of samples P7/H100–500 were similar to those of sample P7. The high-resolution SEM images and nanopore size distribution were evaluated to determine the effects of hydrogen peroxide addition on the nanopore structure (Figure 3). The average pore size of sample P7 (7 wt% phosphoric acid anodizing treatment with no hydrogen peroxide) was 27.54 nm, whereas the average pore sizes in samples P7/H100, P7/H250, and P7/H500 increased to 29.9, 35.13, and 49.77 nm, respectively. Although the difference in pore size between samples P7/H100 and P7/H250 was small (~2–6 nm), the pore size of sample P7/H500 (highest concentration of hydrogen peroxide) was almost double that of sample P7. On the other hand, the average pore sizes in every sample without addition of hydrogen peroxide were similar, regardless of the phosphoric acid concentration as shown in Figure S5 (26.58 nm for P5, 27.54 nm for P7, and 28.97 nm for P15). From these results, it was concluded that the size of the micropits is dependent on the phosphoric acid concentration, while the nanopore size distribution is affected by the amount of the hydrogen peroxide added during the anodizing treatment.

The formation of pores and increase in pore size caused by the addition of hydrogen peroxide were investigated based on the anodizing mechanism [36,37], which has been discussed thoroughly in the Supplementary Materials.

**Figure 4:**
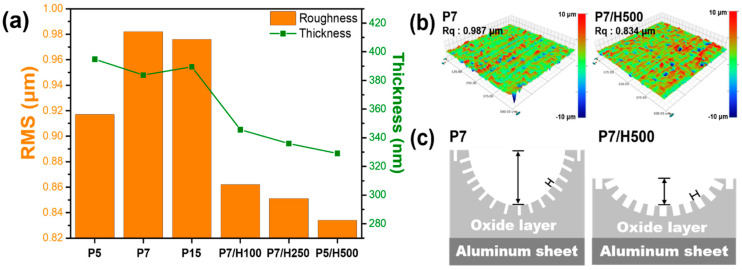
(**a**) Surface roughness and oxide layer thickness of the anodized aluminum, (**b**) surface 3D profile images of the anodized aluminum, and (**c**) illustrations of anodized aluminum surface of sample P7 and P7/H500.

An Alpha-Step profiler was used to measure the surface roughness of the samples treated using various concentrations of phosphoric acid and hydrogen peroxide, because the anodized aluminum surface structure can impact its adhesive performance with the polymer. The root mean square (RMS) roughness and oxide layer thickness, as measured from side-view SEM images, are presented in Figure 4a and Table 1. The RMS roughness increased with increasing phosphoric acid concentration; sample P7 exhibited RMS roughness value of 0.982 μm. Phosphoric acid created an aggressive anodizing environment, thereby increasing the etching rate and producing a rough surface. However, contrary to expectations, the P15 sample recorded a similar RMS roughness value (0.976 μm). During the anodization process, air bubbles are generated, creating a porous oxide layer as they escape. Once this oxide layer reaches a certain thickness, it becomes too thick to allow further air bubble generation. If the electrochemical reaction continues under these conditions, the branches forming the pores are damaged, leading to the collapse of the porous structure and a reduction in film thickness. This process repeats, eventually stabilizing the thickness with minimal deviation over time. The slightly lower roughness observed at P15 was likely due to the collapse of the pore structure at this stage, temporarily smoothing the surface and thus reducing the roughness value relative to expectations.

A sharp decrease in roughness occurred when hydrogen peroxide was added; the RMS roughness value of sample P7/H500 (0.834 μm) was substantially lower than that of sample P7 (0.982 μm). Further, 3D profile images confirmed that the roughness of sample P7/H500 was lower than that of P7 (Figure 4b). The 3D profile images of all other samples are depicted in Figure S6. The decrease in roughness was accompanied by a decrease in the thickness of the aluminum oxide layer (Figure 4a and Table 1). In particular, the oxide layer thickness decreased from 383 nm in sample P7 to 329 nm in sample P7/H500. The decreases in the surface roughness and aluminum oxide thickness were attributed to the increased dissolution of the aluminum oxide layer when hydrogen peroxide was added. The analyses of the anodized aluminum surface indicate that the addition of hydrogen peroxide induced the dissolution of the aluminum oxide layer, thereby leading to decreases in roughness and thickness, and an increase in the size of the nanopores while maintaining the size of the micropits (Figure 4c).

### 3.2. Analyses of Anodized Aluminum Oxide Layer

**Figure 5:**
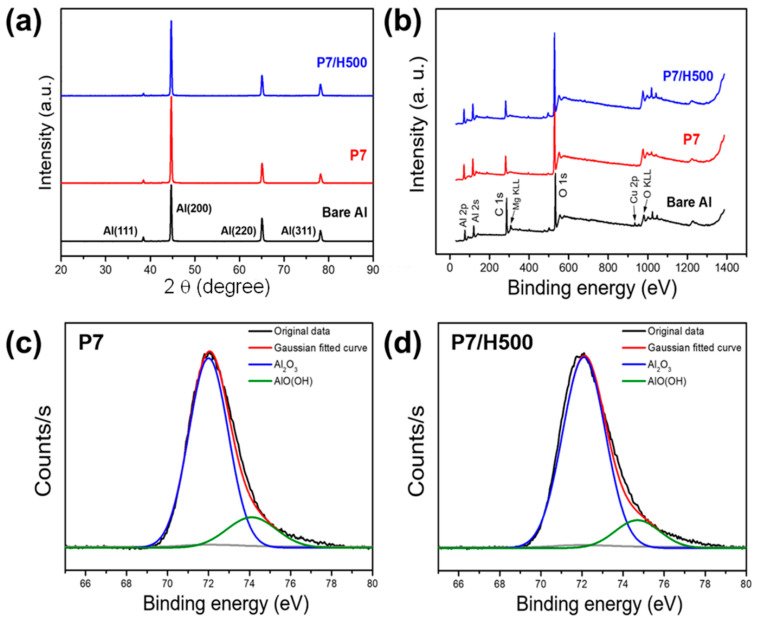
(**a**) XRD patterns and (**b**) XPS survey spectra of untreated aluminum, P7, and P7/H500. High resolution Al 2p XPS spectra of (**c**) P7 and (**d**) P7/H500.

XRD patterns of the aluminum oxide layer are shown in Figure 5a. Before anodizing the aluminum surface, the XRD pattern exhibited characteristic aluminum oxide peaks associated with the (111), (200), (220), and (311) planes at scattering angles (2θ) of 39°, 45°, 65°, and 79°, respectively. The XRD analysis in this study indicated that no crystals were formed during the anodization of the amorphous aluminum oxide layer, which was confirmed by the low intensity of the (111) peak and the pronounced (200) peak after anodizing. Structural analysis of an anodized aluminum oxide layer using XRD as reported by Bara et al. exhibited a more intensive diffraction pattern at ~20–45°, which is characteristic of amorphous aluminum oxide [38]. Another study reported that an amorphous anodic film exhibited a low-intensity (111) peak when anodized in a phosphoric acid solution at various voltages [39]. Both studies demonstrated that the crystallinity of the aluminum was not influenced by anodization.

XPS survey spectra of bare Al, P7, and P7/H500 samples are shown in Figure 5b. The peaks corresponding to Al, O, Cu, C, and Mg were observed in the bare Al sample. The low proportions of the O 1s and Al 2p peaks of this sample, corresponding to 31.54 and 17.13 at%, respectively, were attributed to the presence of impurities. In the case of sample P7, the peaks attributed to impurities such as Cu and Mg were not observed and the proportions of the O 1s and Al 2p peaks increased to 45.42 and 25.24 at%, respectively. Similarly, the contents corresponding to O 1s and Al 2p in sample P7/H500 were 44.16 and 23.94 at%, respectively. These findings indicated that impurities were removed during pretreatment with NaOH and H_2_SO_4_, and the aluminum oxide layer was formed during anodization. SEM also confirmed the removal of impurities (Figure S3), where sequential SEM images were captured after pretreatment with the NaOH and H_2_SO_4_ solutions. In particular, NaOH etching removed the portions of the natural oxide layer while simultaneously producing a rough surface, and H_2_SO_4_ removed the thin oxide layer and impurities that remained after etching with NaOH. However, submicron-sized pits remained on the smooth aluminum surface after H_2_SO_4_ etching, attributed to the high roughness of the NaOH-treated surface.

The deconvoluted Al 2p binding-energy XPS spectra of samples P7 and P7/H500 confirmed the presence of aluminum oxide (Al_2_O_3_) and aluminum oxide hydroxide (AlO(OH)) at binding energies of 72.0 and 74.7 eV, respectively (Figure 5c,d). AlO(OH) is also expressed as Al_2_O_3_*H_2_O, and considered as the precursor of aluminum oxide [35]. The atomic percentages of Al_2_O_3_ in samples P7 and P7/H500 were 86.74 and 83.30 at%, respectively, while those of AlO(OH) are 13.26 at% and 16.71 at%, respectively, demonstrating that the atomic percentage of Al_2_O_3_ ultimately decreased by 3.4 at% with the addition of H_2_O_2_. The deconvoluted O 1s binding-energy XPS spectra of two identical samples are shown in Figure S7. The presence of Al_2_O_3_ and AlO(OH) was confirmed at binding energies of 529.0 and 531.7 eV, respectively, and the atomic percentage of Al_2_O_3_ in sample P7/H500 decreased by 3.39 at% compared to that in sample P7, exhibiting the same trend from the Al 2p data. From the deconvoluted XPS spectrum analyses of each specimen, we confirmed that the addition of hydrogen ions (hydrogen peroxide) increased the electrochemical dissolution of the Al_2_O_3_ layer, leading to the formation of a larger proportion of AlO(OH) compared to treatments that use only phosphoric acid. Water contact angle experiments further confirmed that the sample P7/H100 sample had a higher AlO(OH) content than the sample P7. The sample P7/H100, which contained a relatively higher amount of hydrophilic AlO(OH), exhibited a smaller water contact angle (10°) than the sample P7 (16°) (Figure S8).

### 3.3. Adhesive Properties of Anodized Aluminum and Polymer Resin

**Figure 6:**
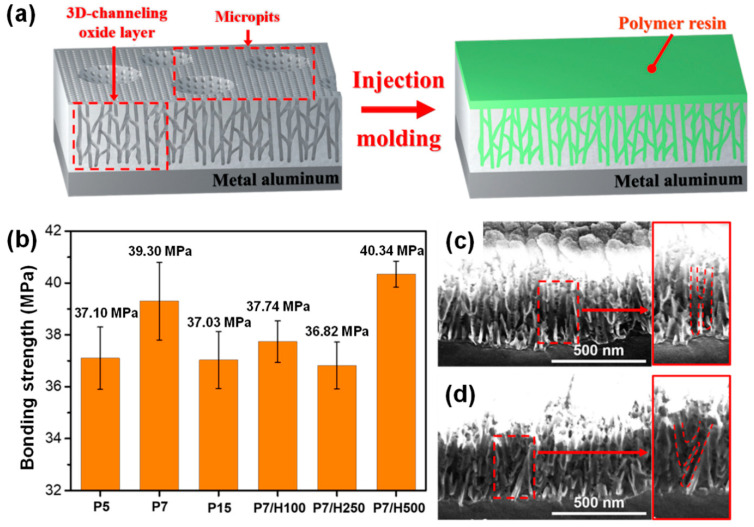
(**a**) Illustration of the injection molding process wherein PBT resin penetrates into the 3D channeling pore structure of the aluminum oxide layer. (**b**) Tensile strength of polymer/metal joint samples with aluminum prepared under various anodization conditions. Cross-section SEM images of (**c**) P7 and (**d**) P7/H500.

PBT resin and anodized aluminum samples were joined using injection molding, where high temperature and pressure enabled the polymer resin to penetrate the porous structure of the oxide layer produced during anodization (Figure 6a). Tensile strength tests were conducted to evaluate the bonding strength of the joined material (Figure S9); the bonding strengths after injection of the polymer resin are shown in Figure 6b.

The bonding strengths of polymer/metal joint samples with P5 and P15 were relatively low at 37.10 with a standard deviation of 1.80 and 37.03 MPa with a standard deviation of 1.63, respectively, while the bonding strength value of the joint sample with P7 was higher by ~2 MPa (39.30 MPa, standard deviation of 2.45) than those of P5 and P15. This was attributed to the difference in the size of the micropits, which has been reported to facilitate the improved penetration of the polymer resin. The micropits formed in sample P5 have diameters of only ~0.2 μm, and the nanopore formation was unstable (Figure 2). Conversely, the low bonding strength of polymer/metal joint sample with P15 was attributed to the very large micropits (>3 μm) caused by excessive etching, which acted as a defect. In contrast, ideal micropits with diameters of ~1 μm were uniformly formed on the aluminum surface of sample P7, thereby achieving a relatively high bonding strength. Thus, the optimal concentration of the phosphoric acid solution tried in this study was 7 wt%, which enhanced the adhesive properties of the polymer/metal joint.

Polymer/metal joint samples with P7/H100, P7/H250, and P7/H500 exhibited bonding strengths of 37.74, 36.82, and 40.34 MPa, respectively. The lower bonding strengths of joint samples with P7/H100 and P7/H250 were attributed to the reduced roughness caused by the addition of hydrogen peroxide. The aluminum surface exhibited a high roughness value after etching with phosphoric acid, however, hydrogen peroxide reduced the contact area at the interface between the aluminum and polymer resin, thereby negatively affecting the adhesive properties. On the other hand, the joint sample with P7/H500 exhibited the highest value of 40.34 MPa with a maximum value of 41.34 MPa (standard deviation of 0.82), owing to the positive effect of hydrogen peroxide on the size of the nanopores, which is the most important factor for determining the adhesive properties of a polymer/metal joint. Xie et al. used a hydrazine (N_2_H_4_) solution to form pores on the surface of aluminum, and the pore size increased during the treatment period [40]. This study indicated that small nanopores (~20 nm) limited the penetration of the molten polymer resin into the oxide layer, whereas larger nanopores (~50 nm) were the most appropriate for polymer resin penetration. The nanopores serve as an entrance for the molten polymer resin, where a larger pore allows for a larger amount of resin within the aluminum oxide layer, thereby leading to increased bonding strength. Although P7/H100 and P7/H250 exhibited an increase of ~2–8 nm in the pore size compared to that of P7, overall adhesive properties of their joint samples decreased because the reduced roughness effect was dominant. In contrast, the pores in P7/H500 nearly doubled in size (49.77 nm), thereby overcoming the decreased roughness. The increased resin penetration in this sample resulted in an outstanding bonding strength of 40.34 MPa. A bonding strength exceeding 40 MPa is considered exceptionally high for polymer/metal joint samples to date (Table S1) [41,42,43,44].

The intrinsic structure of the well-ordered linear pores in the aluminum oxide layers, commonly formed during anodization with oxalic acid, led to weak physical adhesion after the penetration of the polymer resin. However, the porous oxide layer formed using the approach proposed in this study exhibited outstanding adhesive properties. This improved strength was attributed to the enhanced penetration of the polymer resin into the aluminum oxide layer with a 3D-channeling pore structure that induced an interlocking effect. The oxide layers containing 3D-channeling pores are visualized in the cross-sectional SEM images of samples P7 and P7/H500 (Figure 6c,d).

Furthermore, the difference in the main factors governing the bonding strength of polymer/metal joint samples with P7 and P7/H500 was evidenced by differences in the error range. Although the high roughness of sample P7 led to a bonding strength of 39.3 MPa, the small degree of resin penetration into the narrow pore size resulted in a wide error range of ±1.5 MPa (Figure 6b). On the other hand, despite its low roughness value, the joint sample with P7/H500 exhibited a higher bonding strength and a narrower error range of ±0.5 MPa. By referencing the roughness data from Figure 4, it can be inferred that the high roughness resulting from anodization in phosphoric acid leads to an unsuitable aluminum surface structure, contributing to larger variations in bonding strength. In contrast, samples treated with anodization in both phosphoric acid and hydrogen peroxide exhibit relatively lower roughness and smaller variations in bonding strength, because of the pronounced interlocking effect with the large amount of the resin in the pores. This suggests that surface roughness significantly influences the final bonding quality.

### 3.4. 3D Printing of Polymer Resin onto Anodized Aluminum

**Figure 7:**
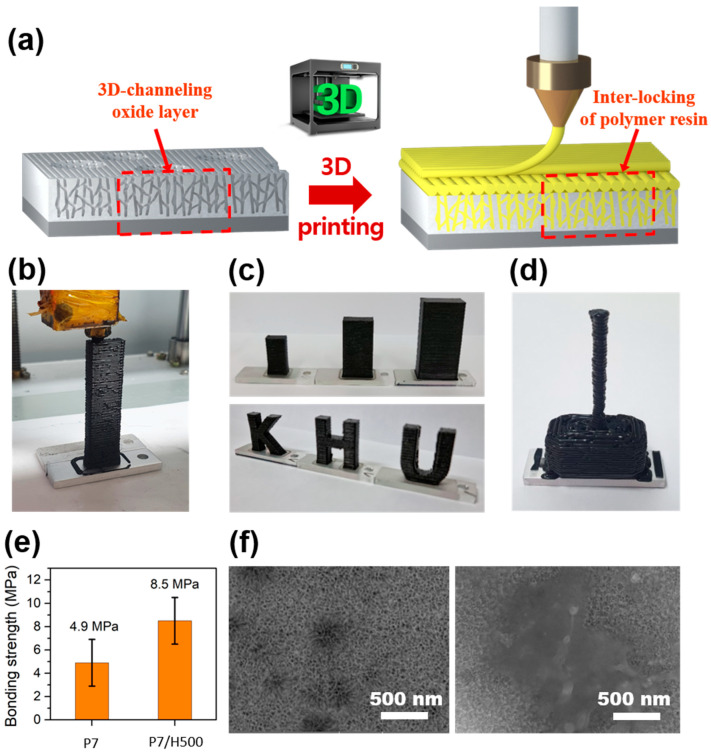
(**a**) Schematic diagram and (**b**) photograph of 3D printing of polymer resin onto the surface of anodized aluminum; (**c**,**d**) photographs of various size and shapes of 3D-printed polymer resin; (**e**) bonding strength of 3D-printed polymer/metal joint samples; (**f**) SEM images of anodized aluminum surface before and after bonding strength test.

3D printing, also known as additive manufacturing (AM), is one of the most efficient molding processes for polymer resins developed recently. This technology allows the creation of complex geometries in a programmable, facile, and flexible manner, without the need for both preparing molds and wasting polymer resin [45]. In this section, a completely new approach was made to join the polymer resin with the anodized aluminum developed in this study through a 3D printing process (Figure 7a). Although the polymer resin was not 3D-printed on the bare aluminum surface even after several attempts (Figure S10), the resin was printed very stably on the anodized aluminum, P7 and P7/H500 (Figure 7b). The results of the contact angle measurements indicated that the surface of bare aluminum was not conducive to contact formation (Figure S8). Additionally, the physical bonding effects achievable through etching and anodization were not present on bare aluminum. Consequently, if the printed polymer experienced a stronger attraction to the nozzle than to the substrate, it detached from the substrate and followed the movement of the nozzle, resulting in a printing failure. Interestingly, the polymer resin was 3D-printed and firmly joined on the anodized aluminum specimen, regardless of the printed area and shape (Figure 7c). More complicated geometric structure (figured the shape of Thor’s hammer firmly bonded to a metal substrate) has been achieved by accurately controlling the movement of the 3D printing nozzle (Figure 7d).

To evaluate the adhesive properties of the 3D-printed polymer resin on the anodized Al samples, a special jig was designed and fabricated (Figure S11). The bonding strength of a 3D-printed polymer/metal joint sample with P7 is relatively low at 4.90 MPa, with a standard deviation of 1.63, while the bonding strength value of the joint sample with P7/H500 was improved to 8.50 MPa with a standard deviation of 3.75 (Figure 7e). Although the bonding strengths were much lower than those of the polymer/metal joint samples manufactured through injection molding (40.34 MPa for P7/H500), it was confirmed that the 3D printing process can join polymer resins to the aluminum surface very quickly, easily, and accurately without the need for heavy molds and equipment. It is worth mentioning that the enhancement in the bonding strength of the 3D-printed polymer/metal joint samples with the addition of hydrogen peroxide (from 4.90 MPa for P7 to 8.50 MPa for P7/H500) was much larger than that of the injection molded polymer/metal joint samples (from 39.30 MPa for P7 to 40.34 MPa for P7/H500). This is presumably because the increase in the size of the nanopores formed by the addition of hydrogen peroxide can be a more critical variable in the case of the polymer/metal joint via the 3D printing process, in which molding (joining) is performed only through the high-temperature melting of a polymer resin without pressure.

Observing the surface of the anodized aluminum from which the 3D-printed polymer resin was peeled off during the bonding strength test, it was visually confirmed that a larger amount of polymer resin remained in the P7/H500 sample than in the P7 sample (Figure S12). Further, SEM images clearly showed that the remarkable amount of 3D-printed polymer resin penetrated the nanopores and micropores on the anodized aluminum surface of the P7/H500 sample (Figure 7f). From this observation, it was also confirmed that the anodized aluminum sample, whose nanopore size increased with the addition of the hydrogen peroxide, is advantageous for polymer resin joining through 3D printing as well as injection molding.

The adhesive properties of the 3D-printed polymer/metal joint could be further improved through, e.g., plasma treatment on the anodized aluminum surface, well-control of the 3D printing conditions, and diversification of the 3D-printed polymer resins [46,47,48,49,50,51]. While the joining strength (8.50 MPa for P7/H500) was much lower than that of conventional injection-molded joining (40.34 MPa for P7/H500) (Figure 7e), 3D printing allows for the creation of polymer/metal joints with various 3D shapes on anodized aluminum substrates without the need for mold manufacturing and injection molding processes. Furthermore, nanostructured surfaces created on aluminum via anodizing can be leveraged to evaluate preliminary potential for diverse industrial applications through 3D printing. Once optimized structures and materials are identified, large-scale production of products via injection molding becomes feasible. Therefore, this process is anticipated to be applicable for advanced or engineered applications involving polymer–metal joints, including durable components for electronic devices, automotive parts, and military equipment.

In addition to the various applications of the anodization process, it is important to develop environmentally friendly manufacturing techniques that adhere to industry standards. While hydrogen peroxide is generally regarded as environmentally benign because it decomposes into water and oxygen, phosphoric acid, which is commonly used in various industrial applications, presents environmental risks. These risks are primarily associated with its potential to cause eutrophication if not properly managed. To mitigate these risks, it is crucial to implement efficient waste treatment processes that can neutralize and safely dispose of phosphoric acid residues. Furthermore, using less concentrated solutions or recycling the anodizing baths can help reduce overall consumption and minimize the environmental burden.

## 4. Conclusions

An anodization technique was applied to generate nanopores on the surface of a widely used commercial aluminum alloy, and PBT was introduced into the nanopore structure of the alloy via injection molding, thereby joining the two distinct materials. The treatment of aluminum with phosphoric acid led to the formation of an aluminum oxide layer with a 3D-channeling pore structure that induced an interlocking effect when the polymer resin was applied. The microstructure of the aluminum oxide layer was dependent on the concentration of phosphoric acid during anodization. When the aluminum was anodized with 7 wt% phosphoric acid, ideal micropits with diameters of ~1 μm were uniformly formed on the aluminum surface. This structure facilitated the penetration of the polymer resin, resulting in a polymer/metal joint with high bonding strength (39.30 MPa). This strength was also attributed to the large polymer/metal interface area because of its high surface roughness. Furthermore, hydrogen peroxide was added to generate additional hydrogen ions during the anodizing process, which promoted the dissolution of the aluminum oxide to increase pore size. The addition of 500 mM of hydrogen peroxide resulted in the largest increase in nanopore size of ~50 nm, and, in turn, an extremely high bonding strength of the polymer/metal joint (40.34 MPa); this measurement had a narrow error range of ±0.5 MPa due to the pronounced interlocking effect with the large amount of resin in the pores. This alternative approach addresses environmental issues associated with the outdated use of Cr(VI)-based anodizing solutions.

Finally, another polymer resin, PLA, was joined onto the anodized aluminum surface through the well-developed 3D printing process. The simple 3D printing could injected the polymer resin into the nanopores as well as the micropores on the anodized aluminum surface, resulting in a polymer/metal joint with bonding strength of 8.50 MPa. In this study, however, the proposed anodization and polymer/metal bonding method demonstrated high bonding strength specifically with the aluminum alloy and polymers like PBT and PLA that were tested. Therefore, the applicability of this method is currently limited to these combinations. To evaluate its broader applicability to other lightweight composites and shape-memory composites [52], further studies are necessary. Further research will focus on experiments on bonding of various metals with carbon and metal composites to assess the generalizability of this approach.

## Figures and Tables

**Table 1:** Root mean square roughness and oxide layer thickness of each sample.

	P5	P7	P15	P7/H100	P7/H250	P7/H500
RMS [μm]	0.917	0.982	0.976	0.862	0.851	0.834
Thickness [nm]	394.78	383.76	389.52	345.69	335.97	329.06

## Data Availability

The original contributions presented in this study are included in the article/Supplementary Material. Further inquiries can be directed to the corresponding authors.

## Supplementary Materials

The following supporting information can be downloaded at: https://www.mdpi.com/article/10.3390/polym17030299/s1, Figures S1 and S2: Bonding strength data; Figures S3–S5: SEM images; Figure S6: Surface 3D profile images; Figure S7: XPS data; Figure S8: Water contact angle; Figure S9–S12: Photo images; Table S1: List of metal-polymer joints. References [53,54,55,56,57,58,59,60,61,62,63,64,65,66,67,68,69,70] are cited in the supplementary materials.
