# Supplementary material for: Improvement of Polymer/Metal Adhesion Using Anodizing Treatment and 3D Printing Process
Source: Polymers (Basel). 2025 Jan 23;17(3):299. doi: 10.3390/polym17030299 (PMC11820388; doi:10.3390/polym17030299)
Supplement: Supplementary file 1 [file polymers-17-00299-s001.zip › polymers-3430285-supplementary.pdf]

Supplementary data

Improvement of polymer/metal adhesion using anodizing treatment and 3D printing process

Seung Wan Ryu [1,†], Dong Hyun Kim [2,†], Wonhwa Lee [2], Jin-Yong Hong [1,3], Young-Pyo Jeon [1,3,*], and Jea Uk Lee [2,*]

[1] *Hydrogen&C1 Gas Research Center, Korea Research Institute of Chemical Technology (KRICT), 141 Gajeong-ro, Yuseong-gu, Daejeon, 34114, Republic of Korea*

[2] *Department of Advanced Materials Engineering for Information and Electronics, Integrated Education Institute for Frontier Science and Technology (BK21 Four), Kyung Hee University, 1732 Deogyeong-daero, Giheung-gu, Yongin-si 17104, Republic of Korea*

[3] *Advanced Materials and Chemical Engineering, University of Science and Technology (UST), 217, Gajeong-ro, Yuseong-gu, Daejeon, 34113, Republic of Korea*

* Correspondence: ypjeon@krict.re.kr (Y.-P.J.); leeju@khu.ac.kr (J.U.L.); Tel.: +82-42-860-7199 (Y.-P.J.); +82-31-201-3655 (J.U.L.)

† These authors contributed equally to this work.

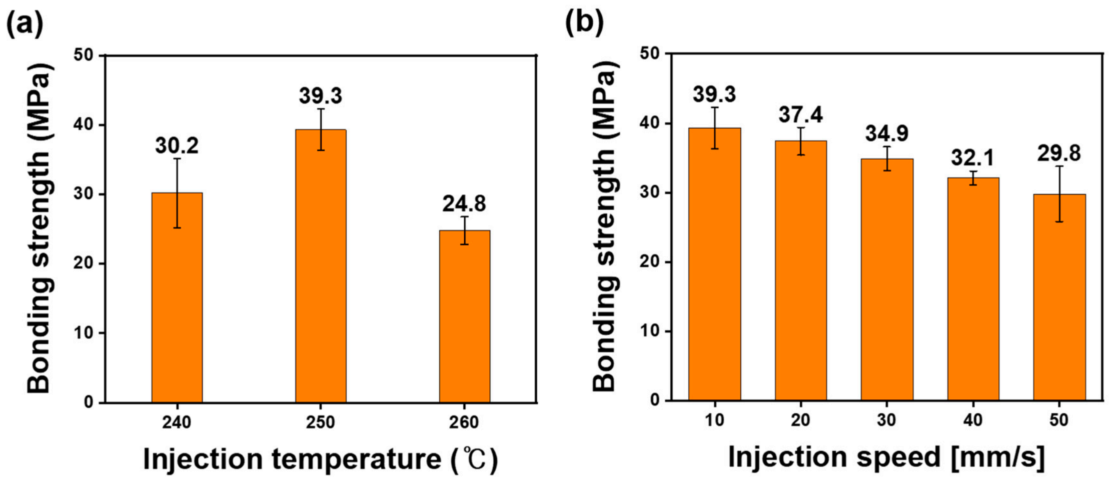

Figure S1. Bonding strength of injection-molded polymer/metal joint samples with P7 (a) as a function of injection temperature at an injection speed of 10 mm/s, and (b) injection speed at an injection temperature of 250 °C.

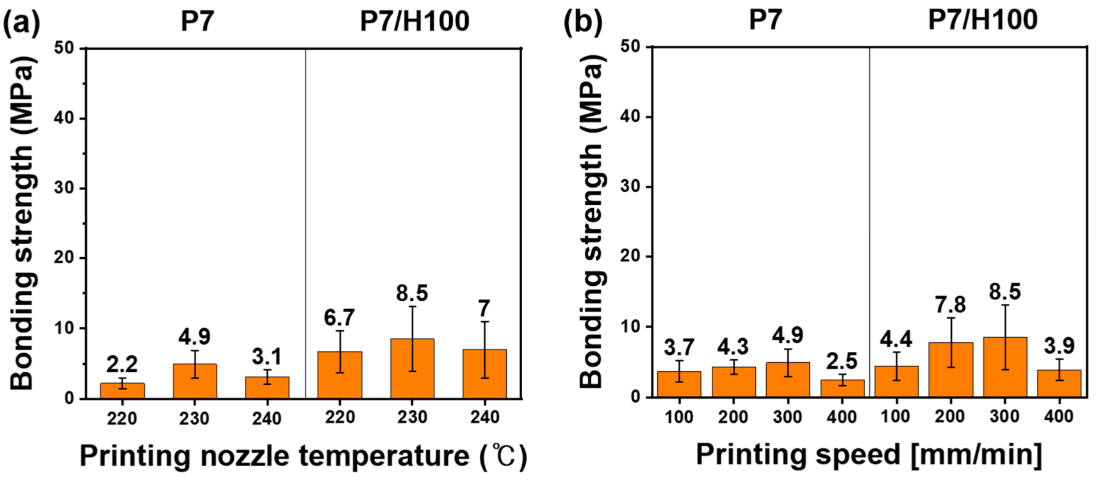

Figure S2. Bonding strength of 3D-printed polymer/metal joint samples as a function of (a) printing nozzle temperature at a printing speed of 300 mm/min, and (b) printing speed at a nozzle temperature of 230 °C.

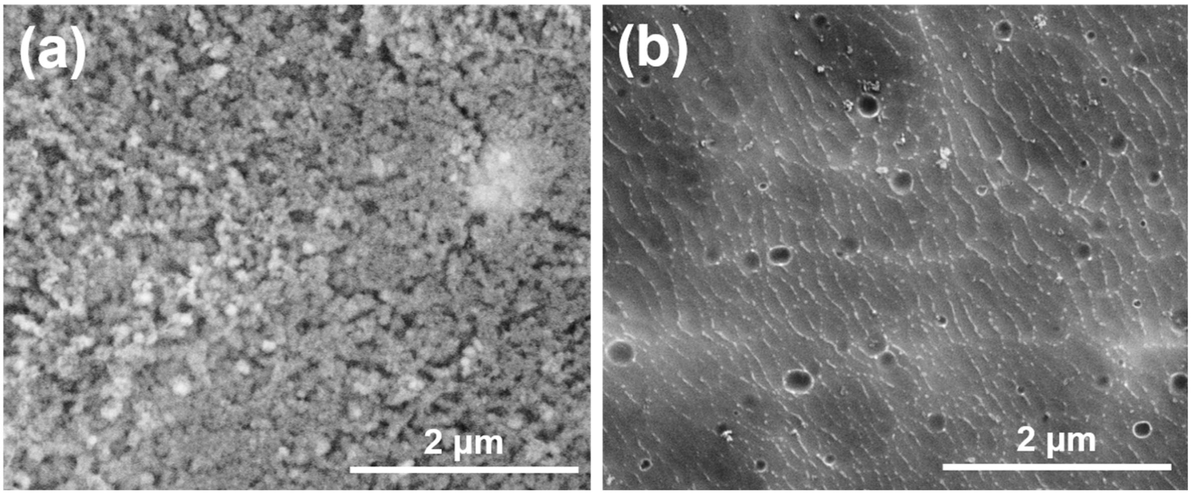

Figure S3. SEM images of aluminum surfaces after sequential pretreatment with (a) NaOH and (b) H_2SO_4.

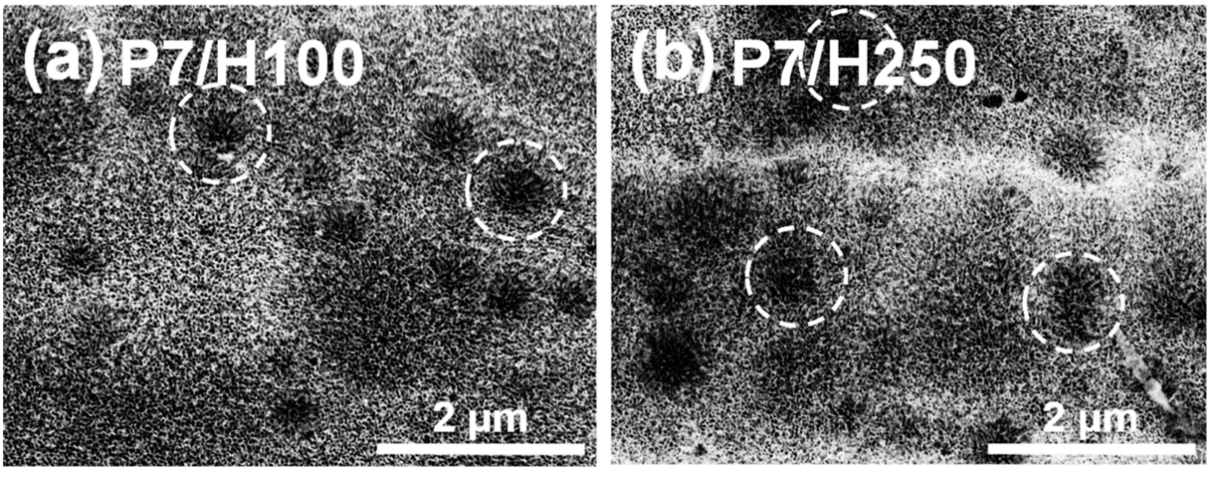

Figure S4. SEM images of anodized aluminum surface with micropits (white dotted circles). (a) P7/H100, (b) P7/H250.

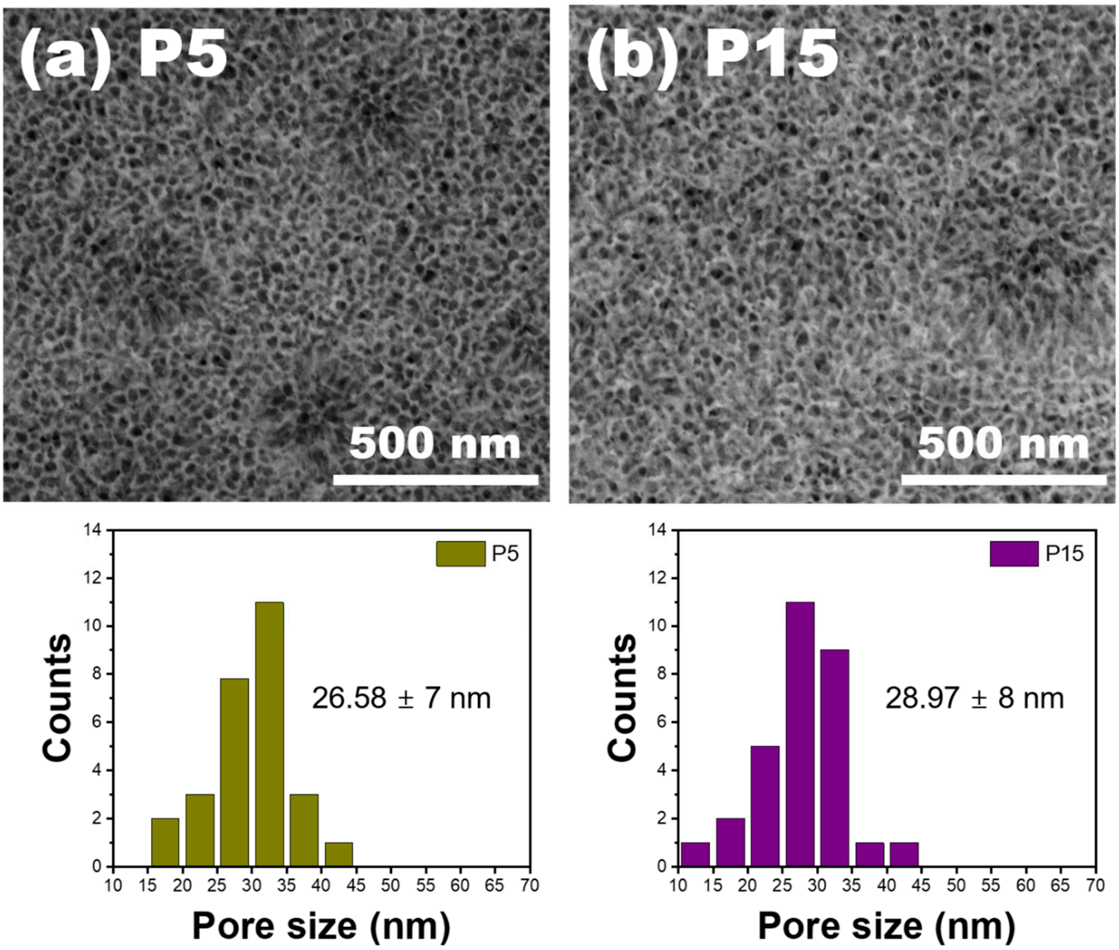

Figure S5. Magnified SEM images of anodized aluminum surface and size distribution of nanopores. (a) P5 and (b) P15.

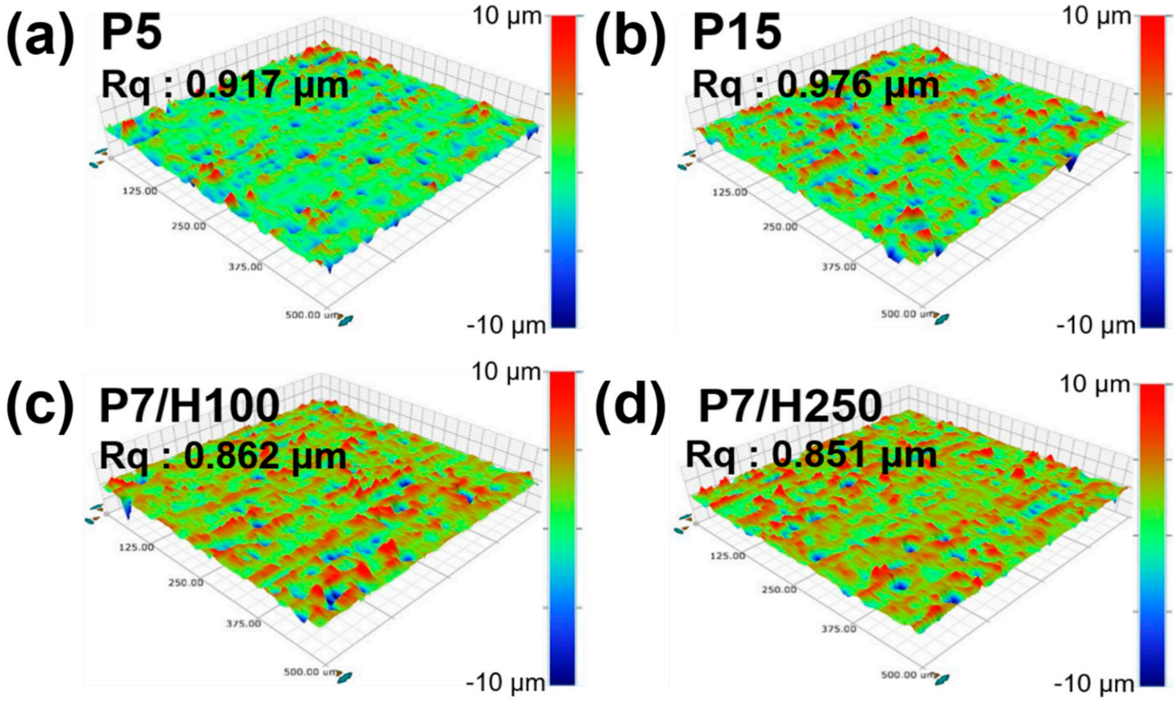

Figure S6. Surface 3D profile images of the anodized aluminum samples. (a) P5, (b) P15, (c) P7/H100, and (d) P7/H250.

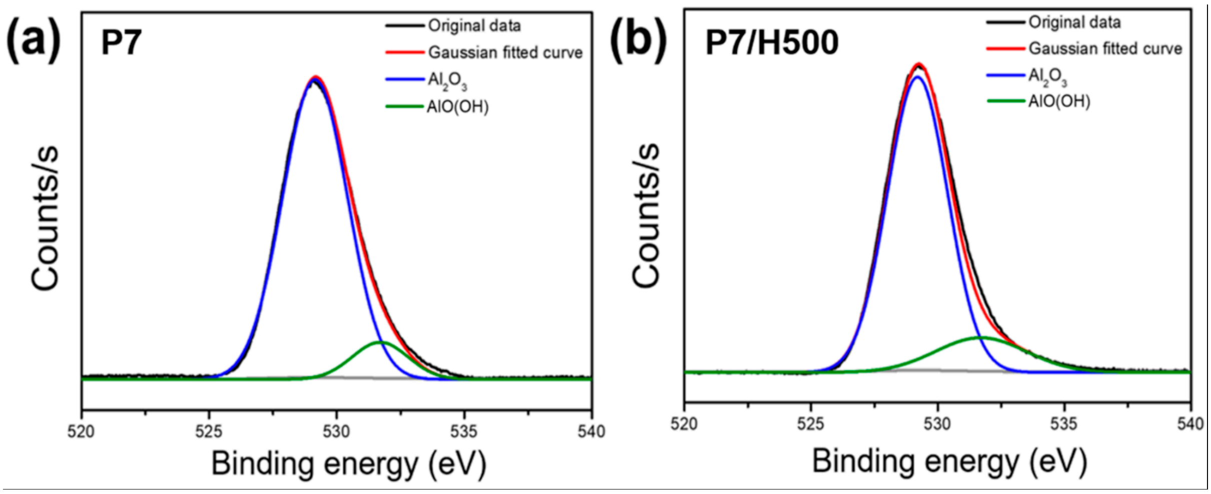

Figure S7. High resolution O 1s XPS spectra of (a) P7 and (b) P7/H500.

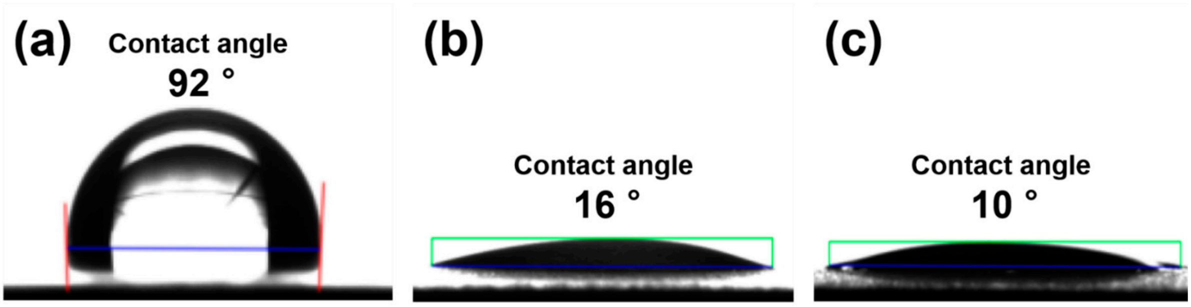

Figure S8. Water contact angle of: a) bare Al, b) P7, c) P7/H500.

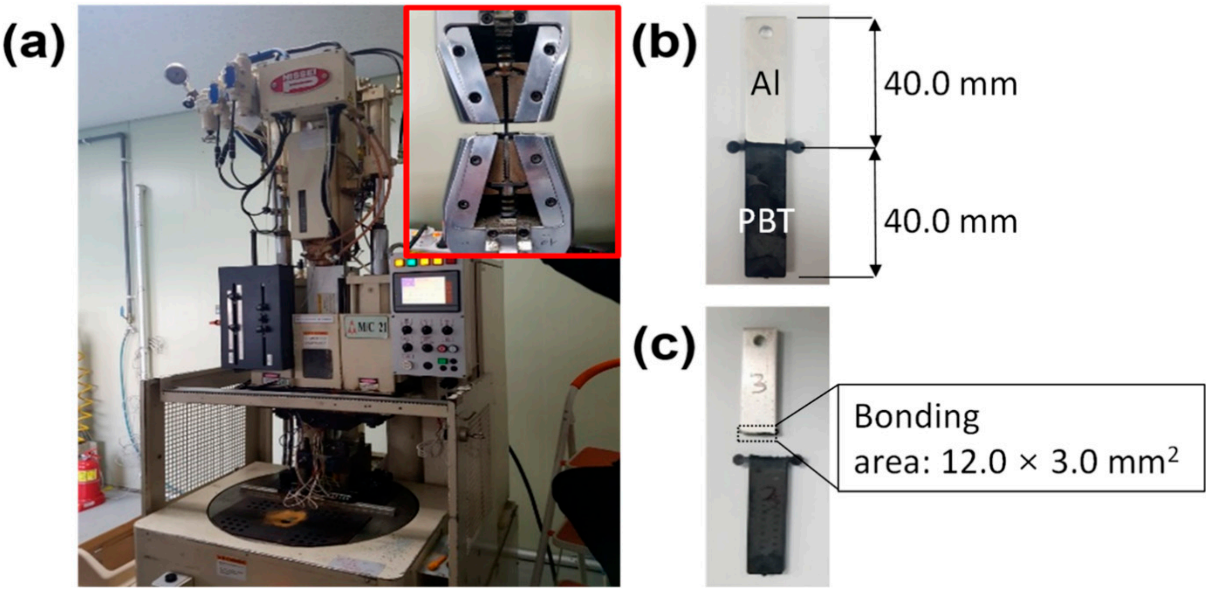

Figure S9. (a) Polymer-metal injection equipment. The inset shows tensile strength measurement equipment. Photographs of polymer–metal joint (b) before and (c) after the measurement of tensile strength.

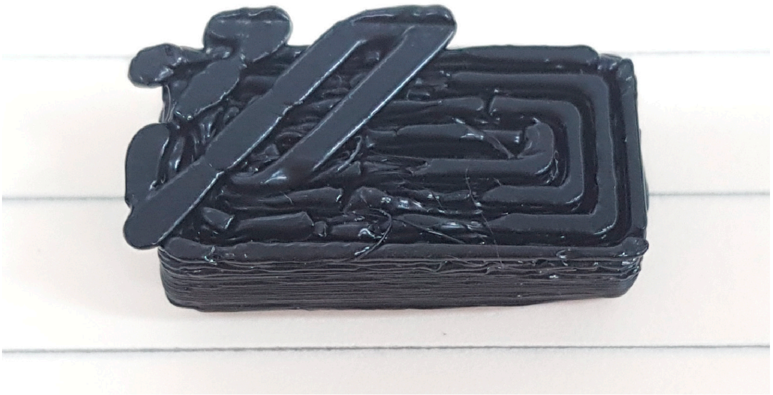

Figure S10. Photograph of 3D printed polymer resin onto the bare aluminum surface that was not anodized.

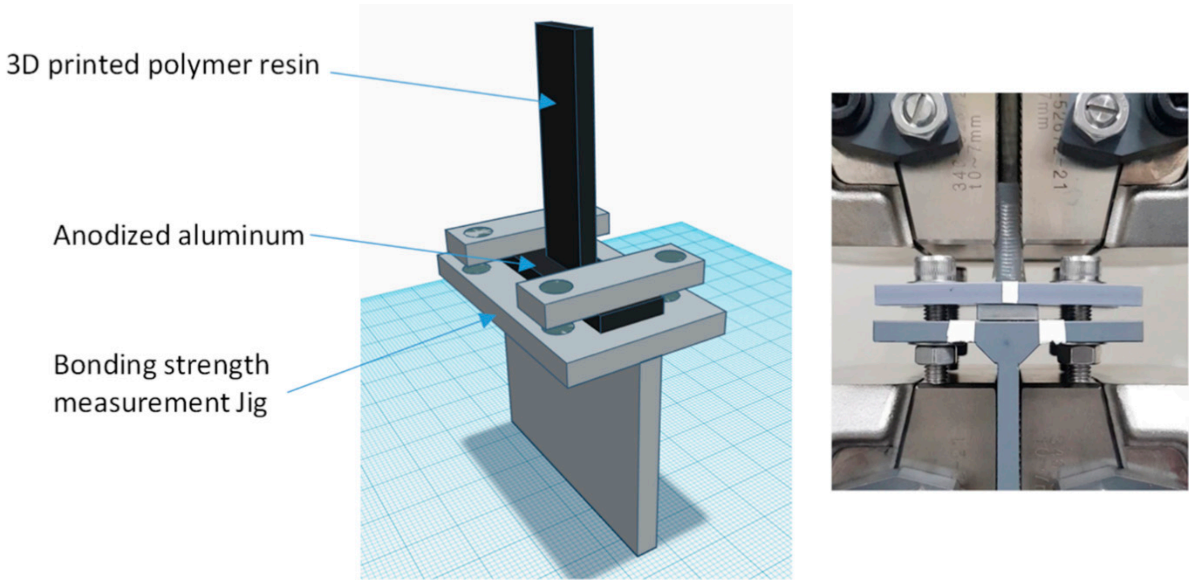

Figure S11. (a) Schematic diagram and (b) photograph of jig for measurement of bonding strength of 3D printed polymer/metal joint sample.

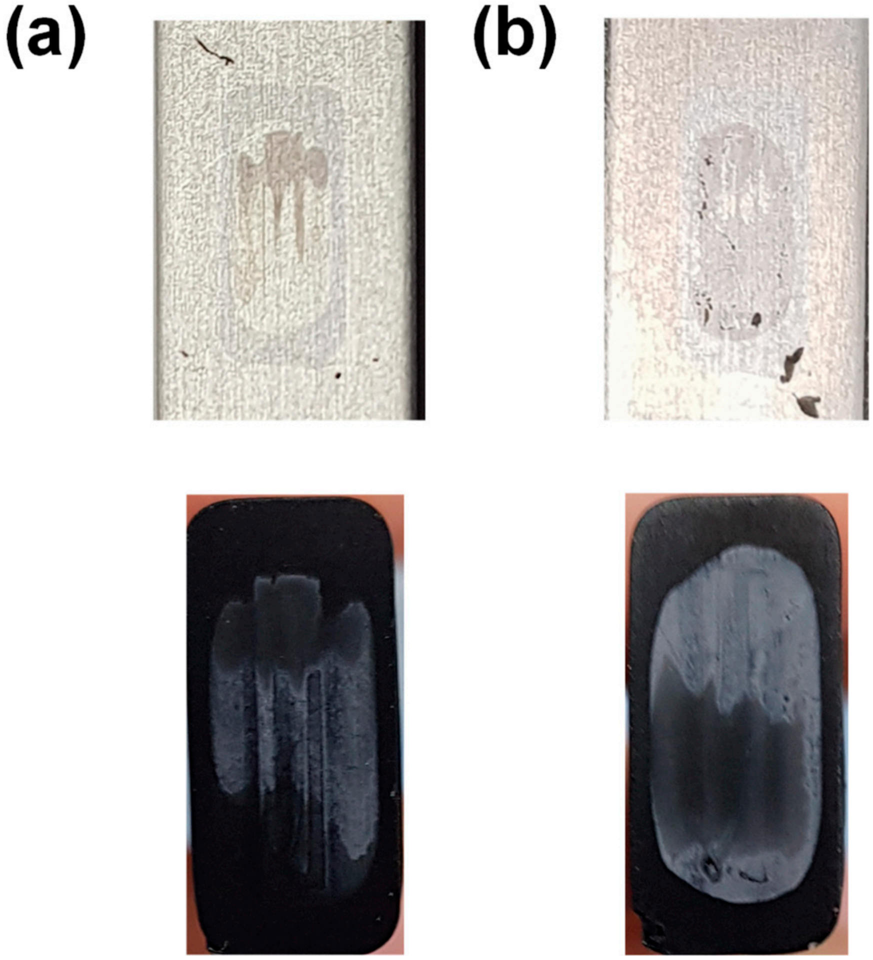

Figure S12. Photographs of failure surface of the anodized aluminum and 3D printed polymer resin after bonding strength test; (a) P7 and (b) P7/H500 samples

Table S1. List of papers describing metal-polymer joint with adhesive properties. Comparisons were drawn on various factors including metal substrates, polymer samples used, pretreatment/ anodizing solution, joint method, bonding strength.

Reference number	Metal	Polymer	Pretreatment/ anodizing solution	Joint method	Bonding strength (MPa)	Note
1	Al alloy	PBT	NaOH, HCl, N_2H_2 / -	Injection molding	23.0	
2	Al alloy	PBT	NaOH, HNO_3 /H_3PO_4	Injection molding	-	
3	Al alloy	PBT	$HClO_4$ /H3PO_4	Injection molding	17.0	
4	Al alloy	PPS	$HClO_4$, C_2H_6O /H_3PO_4	Injection molding	24.8	
5	Al alloy	Nitrile butadiene rubber	H_2SO_4, $H_2C_2O_4$ /H_2SO_4, $H_2C_2O_4$, $C_3H_6O_3$	Injection molding	4.2	
6	Al alloy	CFRTP	NaOH, HNO_3 / H_2SO_4	Ultrasonic vibration-assisted adhesive bonding	20.0	
7	Al alloy	PPS	NaOH, H_2SO_4 / $H_2C_2O_4$, H_3PO_4	Two step anodization	35.0	
8	Al alloy	PA6	SiO_2 /H_3PO_4	Micro-nano hot-press technology	8.24	
9	Al alloy	CFR-PA	- / H_2SO_4	Mechanical blasted	28.0	
10	Al alloy	CFRTP	Picosecond laser processing / H_2SO_4	Laser joining	21.0	
11	Al alloy	Modified PP	- / $H_2C_2O_4$, H_3PO_4	Hot press molding	10.05	
12	Ti-6Al-4V alloy	-	- /HF, H_3PO_4	HA coating	20.0	
13	Al alloy	CFRP	- / H_3PO_4	Fiber metal laminates(FMLs)	23.1	
14	Al alloy	CFRTP	Plasma treatment /-	Expoy bonding	34.3	
15	Al alloy	Polyamide-based thermoplastic adhesive film	- / H_3PO_4	-	17.4	
16	Al alloy	-	- / H_3PO_4	Hot press molding	20.9	Epoxy addition
17	Al alloy	PPS	- / H_2SO_4, H_3PO_4, $H_2C_2O_4$	Injection molding direct joining	21.2	
18	Al alloy	Fiber reinforced composite	- / Tartaric-sulfuric acid	Salt-fog exposition	10.6	

19	Al alloy	-	- / $Na_4P_2O_7$, H_2O, Na_2SiO_3, KOH	Plasma Electrolytic Oxidation	35.89	
20	Ti-25Nb-25Ta alloy	-	- / $C_2H_4(OH)_2$	-	-	Unmeasurable (too weak)
21	Al alloy	CFRP	NaOH, HNO_3 / H_2SO_4, $H_2C_2O_4$	Epoxy based	23.0	
22	Al alloy	PPS	Hydrazine-based chemical treatment	Injection molding	45.0	
This work	Al alloy	PBT	H_3PO_4/H_2O_2	Injection molding	40.34	
This work	Al alloy	PLA	H_3PO_4/H_2O_2	3D printing	8.50	

The formation of pores and increase in pore size caused by the addition of hydrogen peroxide were investigated based on the anodizing mechanism [22,23].

$H_3PO_4 \leftrightarrows H_2PO_4^- + H^+$ (S1)

Anode: $2H_2O\ (l) \rightarrow O_2\ (g) + 4H^+\ (aq) + 4e^-$ (S2)

Cathode: $2H^+\ (aq) + 2e^- \rightarrow H_2\ (g)$ (S3)

Subsequently, the growth of the oxide layer is described as

$Al + 3H^+ \rightarrow Al^{3+}$ (S4)

$Al^{3+}\ (aq) + 3OH^-\ (aq) \rightarrow Al(OH)_3\ (s)$ (S5)

$2Al(OH)_3\ (s) \rightarrow Al_2O_3\ (s) + 3H_2O\ (l)$ (S6)

The dissolution of the oxide layer is described as

$Al_2O_3\ (s) + 6H^+\ (aq) \rightarrow 2Al^{3+}\ (aq) + 3H_2O\ (l)$ (S7)

At high concentrations, phosphoric acid dissociates into hydrogen ions and dihydrogen phosphate (Equation (S1)). When both electrodes are immersed in the phosphoric acid electrolyte and current is applied, water molecules at the anode (aluminum sheet) split to produce oxygen, electrons, and hydrogen ions (Equation (S2)). Hydrogen ions and electrons react at the cathode (graphite sheet) to produce hydrogen gas (Equation (S3)). The hydrogen ions—formed from the decomposition of phosphoric acid and water—react with aluminum to form aluminum hydroxide ($Al(OH)_3$), which is ultimately converted to aluminum oxide (Equations (S4), (S5), and (S6)). The aluminum oxide layer subsequently reacts with hydrogen ions and undergoes dissolution (Equation (S7)).

The addition of hydrogen peroxide leads to the increase of hydrogen ion concentration.

$$H_2O_2\,(l) \rightarrow O_2\,(g) + 2H^+\,(aq) + 2e^- \qquad \text{(S8)}$$

Consequently, the increased hydrogen ion concentration promotes the dissolution reaction (Equation (S7)). Thus, samples treated with phosphoric acid and hydrogen peroxide exhibited larger pores than those treated exclusively with phosphoric acid.

References

1. Xie, Y.; Zhang, J.; Zhou, T. Large-area mechanical interlocking via nanopores: Ultra-high-strength direct bonding of polymer and metal materials. Appl. Surf. Sci. 2019, 492, 558–570.

2. Xu, D.; Yang, W.; Li, X.; Hu, Z.; Li, M.; Wang, L. Surface nanostructure and wettability inducing high bonding strength of polyphenylene sulfide-aluminum composite structure. Appl. Surf. Sci. 2020, 515, 145996.
3. Gong, N.; Wang, B.; Wang, Y.; Li, X.; Lin, W.; Fu, S.; Chu, X. Effect of metal surface state on injection joining strength of aluminum-rubber composite part. J. Manuf. Process. 2020, 49, 365–372.
4. Lin, W.; Li, X.; Dong, W.; Zhao, Y.; Li, M.; Wang, Y. Ultrahigh bonding strength and excellent corrosion resistance of Al-TPU hybrid induced by microstructures and silane layer. J. Mater. Process. Technol. 2021, 296, 117180.
5. Kadoya, S.; Kimura, F.; Kajihara, Y. PBT–anodized aluminum alloy direct joining: Characteristic injection speed dependence of injected polymer replicated into nanostructures. Polym. Test. 2019, 75, 127–132.
6. Wang, H.; Hao, X.; Yan, K.; Zhou, H.; Hua, L. Ultrasonic vibration-strengthened adhesive bonding of CFRP-to-aluminum joints. J. Mater. Process. Technol. 2018, 257, 213–226.
7. Lee, S.; Yashiro, H.; Kure-Chu, S. Effect of Silane Coupling Treatment on the Joining and Sealing Performance between Polymer and Anodized Aluminum Alloy. Korean J. Mater. Res. 2021, 31, 3.
8. Mrzljak, S.; Trautmann, M.; Wagner, F.; Walther, F. Influence of Aluminum Surface Treatment on Tensile and Fatigue Behavior of Thermoplastic-Based Hybrid Laminates. Mater. 2020, 13, 3080.
9. Ye, Y.; Zou, Q.; Xiao, T.; Jiao, J.; Du, B.; Liu, Y.; Sheng, L. Effect of Interface Pretreatment of Al Alloy on Bonding Strength of the Laser Joined Al/CFRTP Butt Joint. Micromachines 2021, 12, 179.
10. Chen, J.; Du, K.; Chen, X.; Li, Y.; Huang, J.; Wu, Y.; Yang, C.; Xia, X. Surface

modification of materials for improved adhesion properties. Appl. Surf. Sci. 2019, 489, 392–402.

11. He, D.; Wang, P.; Liu, P.; Liu, X.; Chen, X.; Li, W.; Zhang, K. Anodic voltage dependence of Ti-6Al-4V substrates and hydroxyapatite coating. J. Nanosci. Nanotechnol. 2019, 19, 5700–5706.
12. Wu, X.; Zhan, L.; Zhao, X.; Wang, X.; Chang, T. Effects of surface pre-treatment and adhesive quantity on interface characteristics of fiber metal laminates. Compos. Interfaces 2020, 27, 829–843.
13. Kim, Y.; Jung, U.; Choi, S.; Jung, Y.; Lee, H.; Kim, J. Effect of plasma gas and Ar incorporation on the shear strength between carbon fiber-reinforced thermoplastic polymer and Al. Compos. Part A 2020, 138, 106041.
14. Sato, K.; Asoh, H.; Yamamoto, H. Effects of nanoporous structure of anodic films on adhesive strength between aluminum alloys and polyamide resin. Mater. Trans. 2021, 62, 1724–1731.
15. Dong, L.; Li, Y.; Huang, M.; Hu, X.; Qu, Z.; Lu, Y. Effect of anodizing surface morphology on the adhesion performance of 6061 aluminum alloy. Int. J. Adhes. Adhes. 2022, 113, 103065.
16. Huang, H.; Sun, M.; Wei, X.; Sakai, E.; Qiu, J. Effect of interfacial nanostructures on shear strength of Al-PPS joints fabricated via injection moulding method combined with anodising. Surf. Coat. 2021, 428, 127896.
17. Di Franco, F.; Fiore, V.; Miranda, R.; Badagliacco, D.; Santamaria, M.; Valenza, A. Influence of anodizing surface treatment on the aging behavior in salt-fog environment of aluminum alloy 5083 to fiber reinforced composites adhesive joints. J. Adhes. 2021, 1–20.
18. Shore, D.; Wilson, J.C.A.; Matthews, A.; Yerokhin, A. Adhesive bond strength of PEO

coated AA6060-T6. Surf. Coat. 2021, 428, 127898.

19. Pereira, B.; Beilner, G.; Lepienski, C.; Souza, G.; Kuromoto, N.; Szameitat, E.; Peng, A.; Lee, J.; Claro, A.; Nugent, M. Scratch-resistant and well-adhered nanotube arrays produced via anodizing process on β-titanium alloy. Mater. Today Commun. 2021, 26, 101947.
20. Cheng, F.; Hu, Y.; Zhang, X.; Hu, X.; Huang, Z. Adhesive bond strength enhancing between carbon fiber reinforced polymer and aluminum substrates with different surface morphologies created by three sulfuric acid solutions. Compos. Part A 2021, 146, 106427.
21. Horiuchi, S.; Terasaki, N.; Itabashi, M. Evaluation of the properties of plastic-metal interfaces directly bonded via injection molding. Manufacturing Rev. 2020, 7, 11.
22. Araoyinbo, A.O.; Rahmat, A.; Derman, M.N.; Ahmad, K.R. Room temperature anodization of aluminum and the effect of the electrochemical cell in the formation of porous alumina films from acid and alkaline electrolytes. Adv. Mat. Lett 2012, 3 273–278.
23. Wang, H.; Wang, H. Thick and macroporous anodic alumina membranes for self-lubricating surface composites. Appl. Surf. Sci. 2005, 249, 151–156
